# Genetic Selection for Voluntary Alcohol Consumption in the Albino Rat

**Published:** 1995

**Authors:** T.-K. Li

**Affiliations:** Ting-Kai Li, M.D., is professor of medicine/biochemistry, School of Medicine, Indiana University, Indianapolis, Indiana

**Keywords:** animal model, AOD consumption, genetics and heredity, selective breeding

**Figure:**
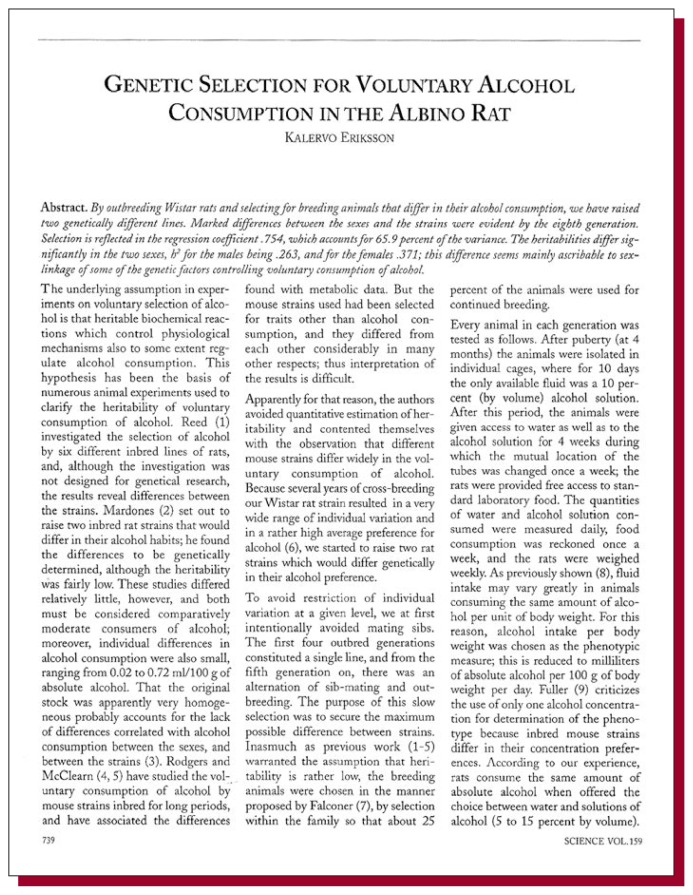
Eriksson, K. Genetic selection for voluntary alcohol consumption in the albino rat. *Science* 159:739–741, 1968.

An important, widely accepted experimental approach to understanding the underlying biology of diseases and their treatment has been the study of animal models. However, among scientists in the alcohol research field, there was doubt even as recently as 15 years ago as to whether studying alcohol consumption behavior in subhuman primates and lower animal species could make a major contribution to the understanding of human alcohol consumption behavior. The principal reason for this skepticism was that voluntary oral consumption of alcohol solutions by animals commonly available in laboratories rarely resulted in supposedly meaningful blood alcohol concentrations unless “unnatural” manipulations, such as limiting the animal’s food intake, and behavioral conditioning procedures are used. Eriksson’s seminal work, as described in this 1968 article, had a major impact in changing this view against using animal models.

Since the 1950’s, inbred strains of rats and mice have been observed to demonstrate different degrees of preference when they have voluntarily consumed alcohol, indicating that this trait is genetically influenced. Building on this knowledge, Eriksson used selective breeding (described below) to develop rat lines whose members showed consistently high and low voluntary alcohol consumption. The procedure he employed was elegant and simple. Rats were given free choice of a 10-percent (volume per volume) solution of pure alcohol in water, a plain water solution, and food over a 4-week period. Rats showing high alcohol preference were mated to start a line (high consumption line, or AA), and rats showing low alcohol preference were mated to start another line (low consumption line, or ANA). This process was repeated over many generations, using care to avoid mating close relatives to lessen the degree of inbreeding. Eventually, a stable difference between the AA and ANA lines of more than five- to sevenfold was obtained.

The continued success of Eriksson’s breeding program over the years encouraged other investigators to replicate these findings. For example, in the United States at this time, there are two pairs of high consumption and low consumption rat lines, the alcohol-preferring P and alcohol-nonpreferring NP lines, and the high-alcohol-drinking HAD and low-alcohol-drinking LAD lines. In Sardinia, Italy, the alcohol-preferring line is called the sP line and the nonpreferring line, the sNP line. Because the four pairs of contrasting lines were developed from parent stocks, the high consumption lines exhibit certain similarities in behavior as well as some differences. The same is true for the low consumption lines. This is consistent with the demonstrated polygenic nature of alcohol-seeking behavior (i.e., the trait appears to be influenced by more than one gene) and supports the finding of genetic subtypes of alcoholism in humans.

There are now hundreds of studies reporting on the behavioral, physiological, biochemical, and genetic associations of alcohol preference in these selected rat lines. At least two important associations of alcohol preference have been found in animals that have clear relevance to human alcohol-consuming behavior. One is the inverse correlation of alcohol sensitivity and preference (i.e., an animal that is highly sensitive to alcohol’s effects will have a low preference for alcohol). Sensitivity is measured by the speed of recovery of impaired function after the administration of a single dose of alcohol and may be attributed to acute alcohol tolerance (i.e., the development of the ability to resist the effects of alcohol within one drinking episode). In the alcohol-preferring rats, for example, this tolerance is present even 10 days after receiving that one dose of alcohol (i.e., they will have a higher resistance to the effects of alcohol 10 days later when they are given another dose). These rats are therefore genetically predisposed to retaining tolerance. In humans, the work of Schuckit (1994) has shown that a lower level of response or sensitivity to alcohol predicts future alcoholism.

Another area in which animal model studies have a clear relevance to human alcohol consumption behavior is the neurobiological basis of alcohol-seeking behavior. Studies in selectively bred rats and other commonly available strains of rats have revealed the involvement of neurotransmitters such as dopamine, serotonin, gamma-aminobutyric acid (GABA), and opioids (and pathways in which these compounds are the primary neurotransmitters) in the reinforcing actions of alcohol. Although no uniform agreement exists as to the relative importance of these pathways (this may, in fact, be attributable to the genetic differences between the models under study), it is worth noting that chemical compounds acting on these pathways can affect the rats’ voluntary alcohol consumption. One such chemical agent is naltrexone, which has now been shown in clinical trials to be effective in some alcoholic patients for reducing alcohol craving. The AA and P rats played important roles in the preclinical development of this treatment.

The full potential of using the selected lines of rats for studying alcoholism-related phenomena, pioneered by Eriksson, is still being realized. For example, with the recent report of a complete genetic linkage map for the laboratory rat (Jacob et al. 1995), the search for the genes underlying alcohol-seeking behavior can now be greatly accelerated. Furthermore, comparing the selected high consumption lines with one another and with other strains of rats under different experimental conditions provides a way to discern different forms of this behavioral trait and to study gene-environment interactions. Both of these endeavors involving animal studies would be relevant for revealing genetic information that may help researchers understand more about human alcohol consumption behavior and alcoholism.
